# Identifying the need for surgical intervention in pediatric bacterial meningitis: single-center experience

**DOI:** 10.3389/fped.2025.1631570

**Published:** 2025-07-16

**Authors:** Merve Boyraz, Servet Yüce, Abdulrahman Özel, Mehmet Tolgahan Örmeci, Süleyman Akkaya, Şeyma Köksal Atiş, Edin Botan

**Affiliations:** ^1^ Department of Pediatrics, Van Training and Research Hospital, Van, Türkiye; ^2^ Department of Public Health, Şırnak Provincial Health Directorate, Şırnak, Türkiye; ^3^ Department of Pediatrics, Division of Pediatric Intensive Care, TC Health Sciences University, Bagcilar Training and Research Hospital, Istanbul, Türkiye; ^4^ Department of Radiology, Van Training and Research Hospital, Van, Türkiye; ^5^ Department of Neurosurgery, Van Training and Research Hospital, Van, Türkiye; ^6^ Department of Pediatrics, Yozgat City Hospital, Yozgat, Türkiye; ^7^ Department of Pediatrics, Division of Pediatric Intensive Care, Van Training and Research Hospital, Van, Türkiye

**Keywords:** meningitis, surgical intervention, empyema, intracranial complications (ICC), cerebrospinal fluid (CSF), pediatric intensive care unit (PICU)

## Abstract

**Background:**

Intracranial complications of bacterial meningitis can arise at any stage and may necessitate neurosurgical intervention. This study evaluates clinical, laboratory, and imaging findings predictive of surgical need in these cases.

**Methods:**

Between 2013 and 2023, 52 pediatric patients with severe neurological symptoms due to bacterial meningitis were admitted to PICU at Van Training and Research Hospital. Patients were classified into two groups: those with intracranial complications (Group 1, *n* = 36) and those without (Group 2, *n* = 16). Group 1 was further divided into those requiring surgery (Group 1B, *n* = 9) and those not (Group 1A, *n* = 27). Statistical analyses were conducted.

**Results:**

Among 52 patients (67.3% male, mean age 76.7 ± 72.0 months), 36 (69.2%) developed intracranial complications, and 9 (17.3%) required surgery. CRP levels were significantly higher in Group 1B (226 mg/dl) than in Group 1A (63 mg/dl) (*p* < 0.001). Significant differences were also found in CSF protein/glucose ratio (*p* = 0.011) and CSF glucose levels (*p* = 0.049). Subdural empyema (SDE) developed in 25 cases, with single-area involvement significantly more frequent in surgical cases (77.8% vs. 12.5%, *p* = 0.012). ROC analysis was performed for CSF protein/glucose, CSF glucose, and serum CRP values.

**Conclusion:**

CRP >150 mg/dl, CSF glucose <6.75 mg/dl, and protein/glucose ratio >18.9 indicate high surgical risk. MRI is recommended for localization, with early neurosurgical consultation and multidisciplinary management for cases with single-area empyema.

## 1 Introduction

Bacterial meningitis (BM) in children is a common and destructive disease of the central nervous system, caused by various pathogens leading to inflammation of the meninges. The main clinical features of the disease include fever, increased intracranial pressure, meningeal irritation, and purulent changes in the cerebrospinal fluid (CSF) (1, 2).

Achieving favorable neurological outcomes in BM relies on early diagnosis, rapid medical treatment, and timely detection of the need for surgical intervention to commence drainage (3–7). A multidisciplinary approach involving a pediatric intensive care specialist, neurosurgeon, neurologist, and physical medicine and rehabilitation specialist is recommended for managing potential neurological complications and surgical needs under intensive care conditions (4, 8).

Throughout the literature, various scoring systems and predictive models have been developed to foresee mortality and sequels in BM prognosis from ancient times to the present (9–11). However, there are no scoring and predictive models specifically designed to identify cases of BM that might require surgical intervention.

The primary objective of this study is to identify early predictive markers—specifically laboratory and CSF parameters obtained at initial admission—that may help clinicians recognize the presence of intracranial complications (ICCs) and estimate their potential severity, including the likelihood of requiring surgical intervention. By highlighting these parameters, we aim to assist physicians in making timely decisions regarding neuroimaging, treatment planning, and intensive monitoring in a pediatric critical care setting.

## 2 Methods

Our study retrospectively examined the records of 52 patients aged 1 month to 18 years, who were diagnosed with meningitis based on clinical, laboratory, and MRI findings between January 2013 and January 2023 at the pediatric intensive care unit.

Patients were included in the study in accordance with WHO meningitis diagnostic criteria (2). The study included patients who were previously healthy without any underlying conditions predisposing to meningitis. Patients were excluded based on the following criteria: (i) patients born prematurely, those with congenital intracranial anomalies, those with ventriculoperitoneal (VP) shunts, patients with immunodeficiency diagnoses, patients with syndromic conditions, cases of meningitis following head trauma, and postoperative brain surgery meningitis cases were excluded. (ii) Additionally, patients for whom CSF findings were not available, those without accessible MRI records or only had CT reports, patients who could not be reached for a physical examination one year after diagnosis, and those with incomplete data were also excluded. Patients whose families refused imaging and lumbar puncture, and all known cases of traumatic lumbar puncture were also excluded from the study.

### 2.1 Patient grouping and definitions

To ensure clarity in our cohort classification, the 52 pediatric patients diagnosed with bacterial meningitis (BM) were categorized based on the presence of intracranial complications (ICC) and surgical intervention status as follows:

• **Group 1**: Patients with one or more intracranial complications (*n* = 36).
  • **Group 1A**: Patients with ICC who did **not require surgery** (*n* = 27).
  • **Group 1B**: Patients with ICC who **required neurosurgical intervention** (*n* = 9).
• **Group 2**: Patients without any identified intracranial complications (*n* = 16).

### 2.2 Definition of intracranial complications (ICC)

Intracranial complications were defined as the development of one or more of the following conditions confirmed through MRI imaging and clinical evaluation: Subdural empyema (SDE), Hydrocephalus, Cerebral thrombosis and/or infarction, Brain abscess, Ventriculitis and Parenchymal shift due to space-occupying lesions. A detailed breakdown of complications and their combinations is presented in Table 1.

**Table 1:** Detailed evaluation of intracranial complications in group 1 patients.

Complication	All group 1 patients (*n* = 36)	Group 1A (*n* = 9)	Group 1B (*n* = 27)
Empyema	25 (69%)	9 (100%)	16 (59%)
*Isolated Empyema*	8 (32%)	1 (11%)	7 (26%)
*Empyema* *+* *Thrombosis*	6 (24%)	1 (11%)	5 (29%)
*Empyema* *+* *Hydrocephalus*	3 (12%)	2 (22%)	1 (4%)
*Empyema* *+* *Ventriculitis*	2 (8%)	0 (0%)	2 (7%)
*Empyema* *+* *Thrombosis* *+* *Abscess* *+* *Infarct*	2 (8%)	1 (11%)	1 (4%)
*Empyema* *+* *Abscess* *+* *Shift*	1 (4%)	1 (11%)	0 (0%)
*Empyema* *+* *Ventriculitis* *+* *Thrombosis*	1 (4%)	1 (11%)	0 (0%)
*Empyema* *+* *Thrombosis* *+* *Hydrocephalus* *+* *Infarct*	1 (4%)	1 (11%)	0 (0%)
*Empyema* *+* *Thrombosis* *+* *Hydrocephalus* *+* *Abscess* *+* *Shift*	1 (4%)	1 (11%)	0 (0%)
Hydrocephalus	4 (11%)	0 (0%)	4 (15%)
Thrombosis	3 (8%)	0 (0%)	3 (11%)
Thrombosis + Infarct	2 (7%)	0 (0%)	2 (7%)
Ventriculitis	1 (3%)	0 (0%)	1 (4%)
Hydrocephalus + Ventriculitis	1 (3%)	0 (0%)	1 (4%)

ICC, intracranial complications.

### 2.3 Surgical indications and procedures performed

Surgical intervention decisions were made by the pediatric neurosurgery team based on a combination of clinical and radiological findings. Objective criteria included the presence of intracranial complications such as subdural empyema, brain abscess, or significant effusion associated with midline shift, cerebral edema, or elevated intracranial pressure. Clinical deterioration despite medical treatment—manifested by altered consciousness, focal neurological deficits, or signs of raised intracranial pressure—also guided surgical decision-making. These criteria were consistently applied across cases to ensure appropriate and timely intervention.

Surgical interventions performed in our study included:

• Decompressive craniotomy (*n* = 4)
• Burr hole drainage (*n* = 3)
• Craniotomy with ventriculoperitoneal (VP) shunt placement (*n* = 2)

Additional procedures such as external ventricular drain (EVD) placement and repeated lumbar punctures were also implemented as part of individualized patient management.

**Figure 1:**
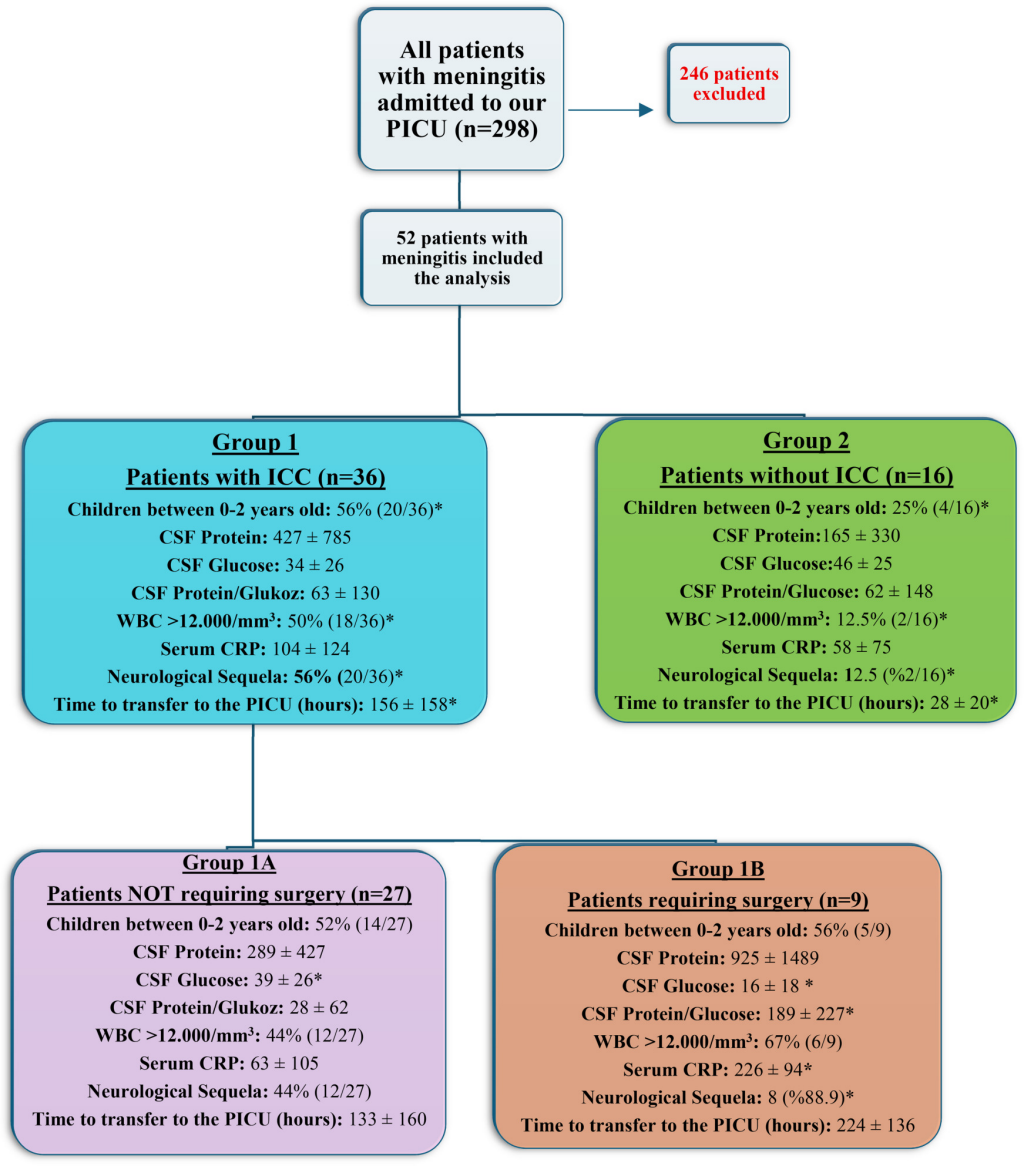
Flowchart of grouping of patients included in our study and the display of key laboratory characteristics of the groups. An asterisk (*) indicates a statistically significant difference compared to the other compared group. ICC, intracranial complications; CSF, cerebrospinal fluid; WBC, white blood cells; CRP, C-reactive protein; PICU, pediatric intensive care unit.

This classification enabled a focused analysis of both the presence of complications and the clinical thresholds necessitating surgical treatment. A visual representation of the grouping is also provided in Figure 1, which includes laboratory markers associated with each subgroup.

### 2.4 Statistical analysis

Data were analyzed using SPSS version 29.0. The statistical parameters analyzed included descriptive statistics (mean, standard deviation) for continuous variables and frequencies and percentages for categorical variables. Comparative analyses were performed using Chi-square tests for categorical variables and independent t-tests or Mann–Whitney U tests for continuous variables, depending on the data distribution. Logistic regression was employed to determine the factors significantly associated with the need for surgical intervention. Receiver operating characteristic (ROC) curves were used to evaluate the diagnostic performance of the significant variables in predicting surgical necessity. Statistical significance was set at *p* < 0.05 for all tests.

## 3 Results

### 3.1 Sociodemographic characteristics of patients

Over a 10-year period, 298 children were admitted to our pediatric intensive care unit with a diagnosis of meningitis. Among them, 52 patients who met the inclusion criteria and had complete records were included in the analysis.

The ages of the patients ranged from 1 month to 18 years, with an average age of 77 ± 72 months. 67% (35/52) of the patients were male, and 33% (17/52) were female.

Intracranial complications developed in 36 of the 52 patients (69%), and emergency neurosurgical interventions were required in 9 of these cases (17%). Neurological deficits were recorded in 44% (23/52) of our patients, with 89% patients (8/9) in the operated group having severe neurological sequelae.

#### 3.1.1 Initial imaging

**Figure 2:**
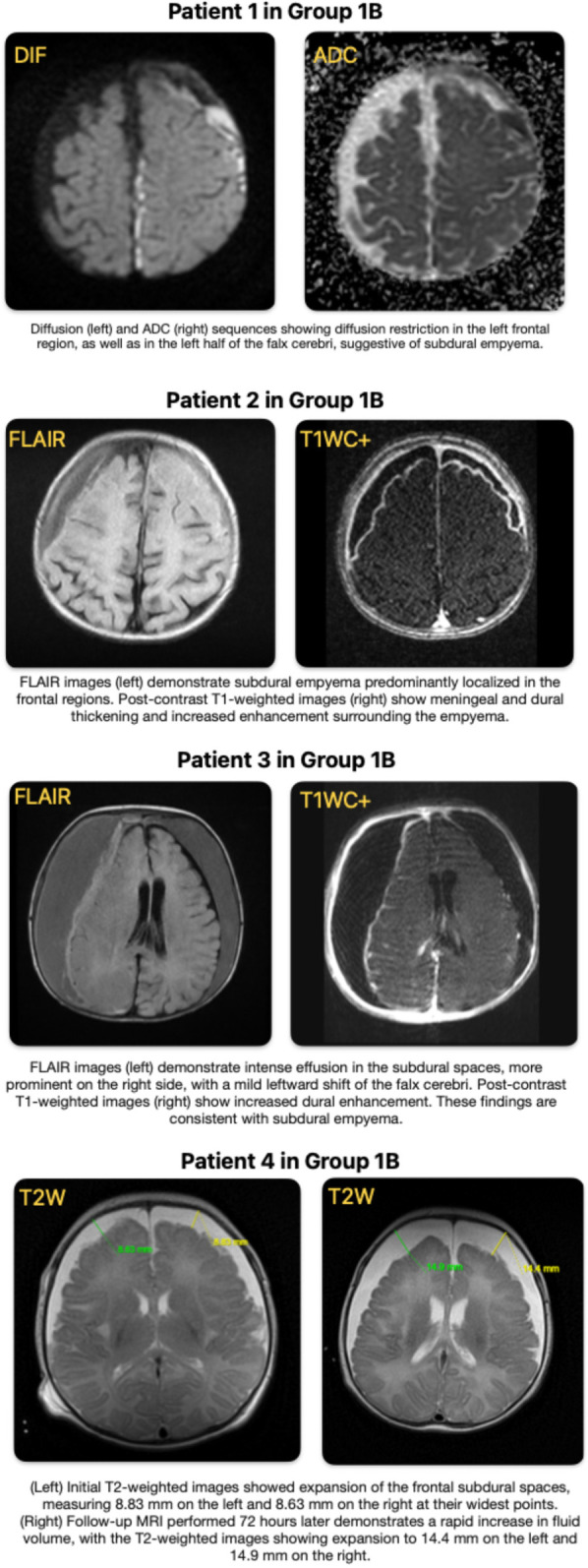
Representative MRI findings of patients with intracranial complications in group 1B.

Among patients diagnosed with intracranial complication (ICC) (Group 1, *n* = 36), 16 patients (44%) had normal cranial CT scans within the first 24 h of admission. These initially unremarkable imaging findings were associated with a delay in obtaining gadolinium-enhanced MRI and initiating appropriate treatment. Of these 16 patients, 14 (87%) subsequently developed neurological sequelae, and 5 (31%) ultimately required surgical intervention. Representative MRI images from four of these patients, along with explanatory annotations, are presented in Figure 2 to illustrate the radiological progression and clinical relevance of delayed diagnosis.

#### 3.1.2 Intracranial complications

The study detailed the complications of the 36 patients who developed intracranial complications. Subdural empyema (SDE) was the most common complication, occurring in 25 of the 36 patients (69%). Of these 25 patients, 8 developed isolated empyema, while 17 developed additional complications alongside the empyema (see Table 1 for details).

#### 3.1.3 Neurosurgical interventions

Among the 9 patients who underwent surgery, a total of 9 procedures were performed: 4 decompressive craniotomies (DC), 3 burr hole surgeries, and 2 craniotomies with ventriculoperitoneal (VP) shunt placements. Each patient underwent a single neurosurgical intervention; no patient received more than one type of surgery. In addition to the operated group, some patients in the non-surgical group required less invasive procedures to manage elevated intracranial pressure. These included repeated lumbar punctures (for CSF drainage) in 2 patients, external ventricular drain (EVD) placement followed by VP shunt in 1 patient, and VP shunt placement alone in another patient.

#### 3.1.4 Mortality

Two patients with intracranial complications were evaluated for surgery and found to have definitive surgical indications. However, both patients experienced severe hemodynamic instability and septic shock, which precluded surgical intervention. Despite maximal medical support, they died before surgery could be initiated. To maintain analytical consistency, these patients were excluded from subgroup analyses.

Detailed descriptions of the complications developed in patients who underwent surgery are presented in Table 1.

### 3.2 Patient grouping

In our study, the 52 patients diagnosed with bacterial meningitis (BM) were divided into two main groups based on the development of intracranial complications (ICC): those who developed complications (36 patients) and those who did not (16 patients). Further, the 36 patients who developed intracranial complications were subdivided into two groups: those who underwent surgery (9 patients) and those who did not require surgical intervention (27 patients). This grouping is illustrated in the flowchart provided in the study documentation.

This classification allowed for a structured analysis of outcomes and interventions based on the presence of intracranial complications and the necessity of surgical procedures, facilitating a clearer understanding of the disease’s impact and the effectiveness of the treatment approaches used.

### 3.3 Characteristics of age groups among the groups

According to the classification above, there was no significant difference in the average ages of children in Group 1 (76 ± 77 months) compared to those in Group 2 (79 ± 61 months) (*p* = 0.699). When children were divided into two age groups, those under 2 years old and those older than 2 years, the proportion of children under 2 years in Group 1 was significantly higher (*p* = 0.041), indicating that the incidence of intracranial complications (ICC) is significantly higher in children between 0 and 2 years old (Table 2). The average ages and age group distributions for children in Groups 1A and 1B were found to be similar (*p* = 0.909 and *p* = 0.847, respectively). However, it was determined that 5 of the 9 patients requiring surgery were between 1 and 6 months old.

**Table 2:** Basic characteristics and laboratory values of patients in groups 1 and 2.

Parameter	Group 1 (Patients with ICC, *n* = 36)	Group 2 (Patients without ICC, *n* = 16)	*P* value
Average age, months	76 ± 77 (1–204)	79 ± 61 (2–192)	0.699^a^
0–2 years, *n* (%)	20 (56%)	4 (25%)	**0**.**041**^b^
>2 years, *n* (%)	16 (44%)	12 (75%)	
Elevated WBC (>12,000 /mm^3^)	18 (50%)	2 (13%)	**0**.**010**^b^
CRP, mg/dl	104 ± 124	58 ± 75	0.172^a^
CSF protein, mg/dl	427 ± 785	165 ± 330	0.279^a^
CSF glucose, mg/dl	34 ± 26	46 ± 25	0.189^a^
CSF protein/glucose ratio	63 ± 130	62 ± 148	0.490^a^
Time from symptom to PICU transfer, hours	156 ± 158	28 ± 20	**<0**.**001**^a^
Neurological sequelae at 1 year, *n* (%)	20 (56%)	2 (13%)	**0**.**004**^b^

ICC, intracranial complications; WBC, white blood count; CRP, C-reactive protein; CSF, cerebro-spinal fluid; PICU, pediatric intensive care unit.

^a^ Independent samples *t* test.

^b^ Pearson chi-square test, significant *p* values are bold.

### 3.4 Differences in laboratory parameters between the groups

When comparing laboratory parameters between Group 1 and Group 2, only the elevation of white blood cells (WBC) defined as >12,000 /mm^3^ was found to be significantly higher in Group 1 (50% vs. 13%, *p* = 0.010). Serum CRP levels (*p* = 0.172), CSF protein (*p* = 0.279), CSF glucose (*p* = 0.189), and CSF protein/glucose ratio (*p* = 0.490) did not show significant differences between the groups.

When comparing laboratory parameters between Groups 1A and 1B, similar levels of WBC elevation were found (*p* = 0.248), however, serum CRP levels were significantly higher in Group 1B (226 ± 94 vs. 63 ± 105, *p* < 0.001). Regarding CSF values, while CSF protein levels were similar between the two groups (*p* = 0.110), CSF glucose levels were significantly lower in Group 1B (*p* = 0.049). The CSF protein/glucose ratio was also significantly higher in Group 1B (*p* = 0.011) (Table 3).

**Table 3:** Basic characteristics and laboratory values of patients in groups 1A and 1B.

Parameter	Group 1A (ICC and NOT requiring surgery, *n* = 27)	Group 1B (ICC and required surgery, *n* = 9)	*P* value
Average age, months	77 ± 77	73 ± 82	0.909^a^
0–2 years, *n* (%)	14 (52%)	5 (56%)	0.847^b^
>2 years, *n* (%)	13 (48%)	4 (44%)	
Elevated WBC (>12,000 /mm^3^)	12 (44%)	6 (67%)	0.248^b^
CRP, mg/dl	63 ± 105	226 ± 94	**<0**.**001**^a^
CSF protein level, mg/dl	289 ± 427	925 ± 1,489	0.110^a^
CSF glucose level, mg/dl	39 ± 26	16 ± 18	**0**.**049**^a^
CSF protein/glucose ratio	28 ± 62	189 ± 228	**0**.**011**^a^
Time from symptom to PICU transfer, hours	133 ± 160	224 ± 136	0.134^a^
Neurological sequelae at 6 months, *n* (%)	12 (44%)	8 (89%)	**0**.**020**^b^

ICC, intracranial complications; WBC, white blood count; CRP, C-reactive protein; CSF, cerebro-spinal fluid; PICU, pediatric intensive care unit.

^a^ Independent samples *t* test.

^b^ Pearson chi-square test, significant *p* values are bold.

### 3.5 ROC analysis for CSF and Serum parameters—relationship to surgical necessity

ROC analyses were conducted to evaluate the predictive power of key CSF and serum parameters for surgical intervention among patients with intracranial complications (*n* = 36).

• **CSF glucose** levels below 6.75 mg/dl were significantly associated with the need for surgery, with an AUC of 0.789 (*p* = 0.016), 60% sensitivity, and 94% specificity.
• A **CSF protein/glucose ratio** >18.9 also showed strong predictive value (AUC: 0.811, *p* = 0.011), with 80% sensitivity and 78% specificity.
• **Serum CRP** levels >150.3 mg/dl demonstrated the highest predictive accuracy (AUC: 0.840, *p* < 0.001), with both sensitivity and specificity at 89%.
• In contrast, **CSF protein** alone was not a statistically significant predictor (AUC: 0.644, *p* = 0.312), though a cut-off of 304.5 mg/dl yielded moderate sensitivity (60%) and specificity (72%).

**Figure 3:**
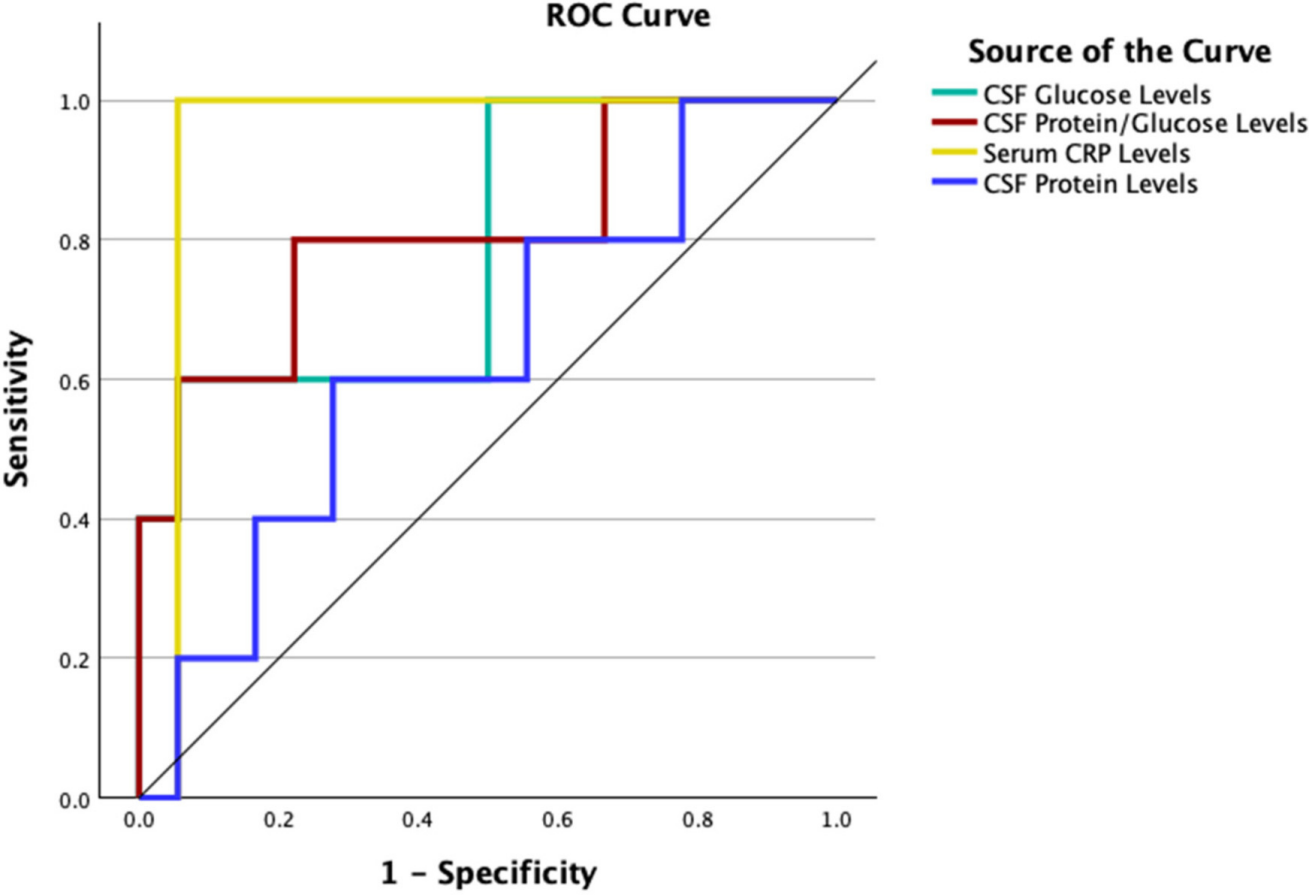
Combined ROC curves demonstrating the predictive performance of key laboratory parameters for surgical intervention in patients with intracranial complications (ICC).

These findings suggest that CRP, CSF glucose, and the CSF protein/glucose ratio may serve as reliable early markers for identifying patients who may require neurosurgical intervention (Figure 3).

Since these three parameters showed significant differences between patients with and without surgical indication in descriptive analyses—and also demonstrated statistically significant predictive value in the ROC analysis—patients who tested positive for all three parameters were further evaluated under the criterion of “3 + parameter positivity.” Accordingly, six patients were identified with simultaneous positivity for CRP, CSF glucose, and the CSF protein/glucose ratio. All of these patients belonged to the group requiring surgical intervention. The presence of 3 + positive parameters was found to have a highly significant predictive power in determining surgical necessity (*p* < 0.001).

### 3.6 Definition of SDE areas and risk of surgery based on involvement

**Figure 4:**
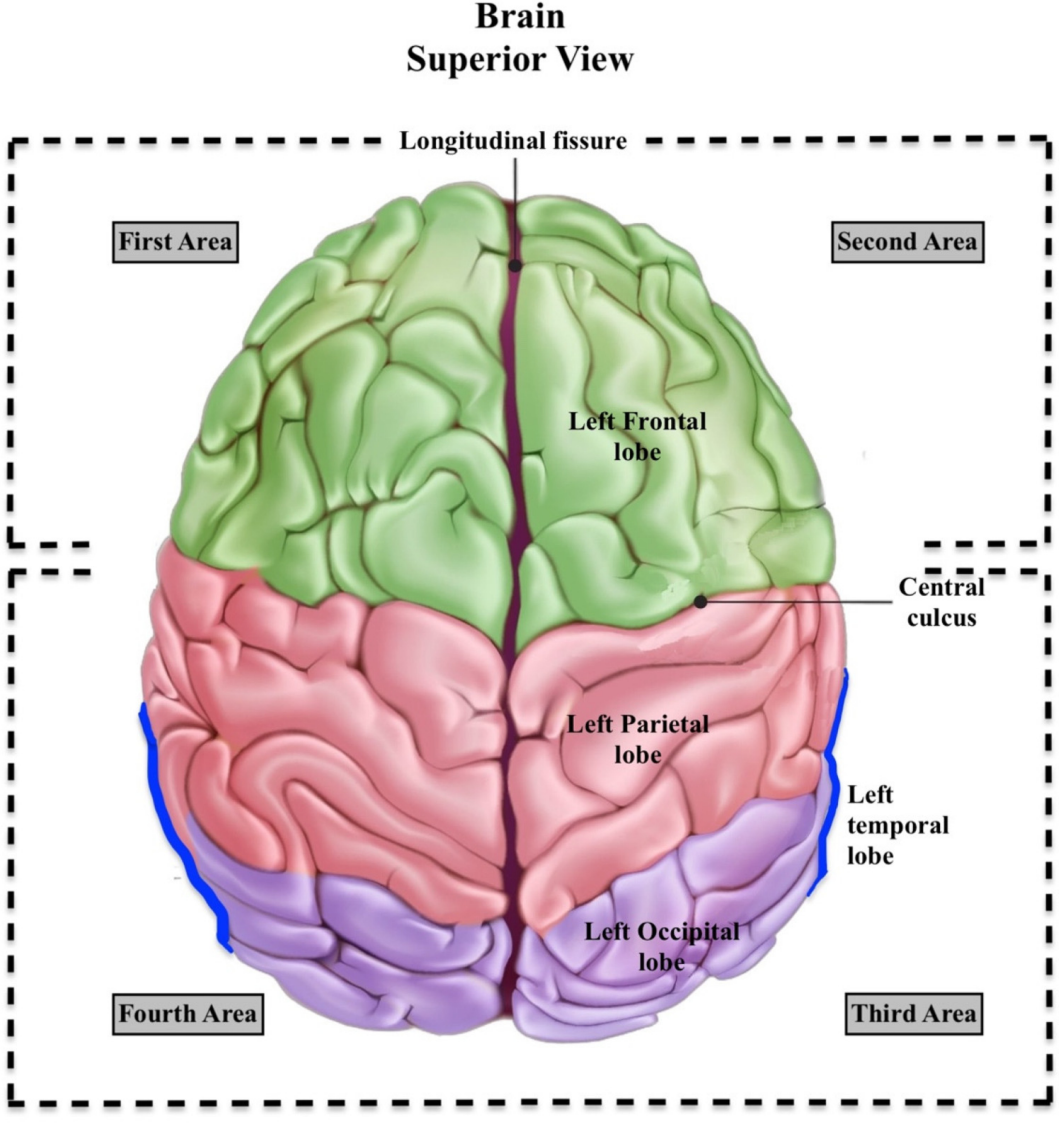
Visual illustration of the first, second, third, and fourth areas. **First area:** Right Frontal Lobe. **Second Area:** Left Frontal Lobe. **Third Area:** Left Occipital Lobe + Left Parietal Lobe + Left Temporal Lobe + Lateral Ventricles’ Occipital Horns (Trigonal neighborhoods), and **Fourth Area:** Right Occipital Lobe + Right Parietal Lobe + Right Temporal Lobe + Lateral Ventricles’ Occipital Horns (Trigonal neighborhoods).

**Figure 5:**
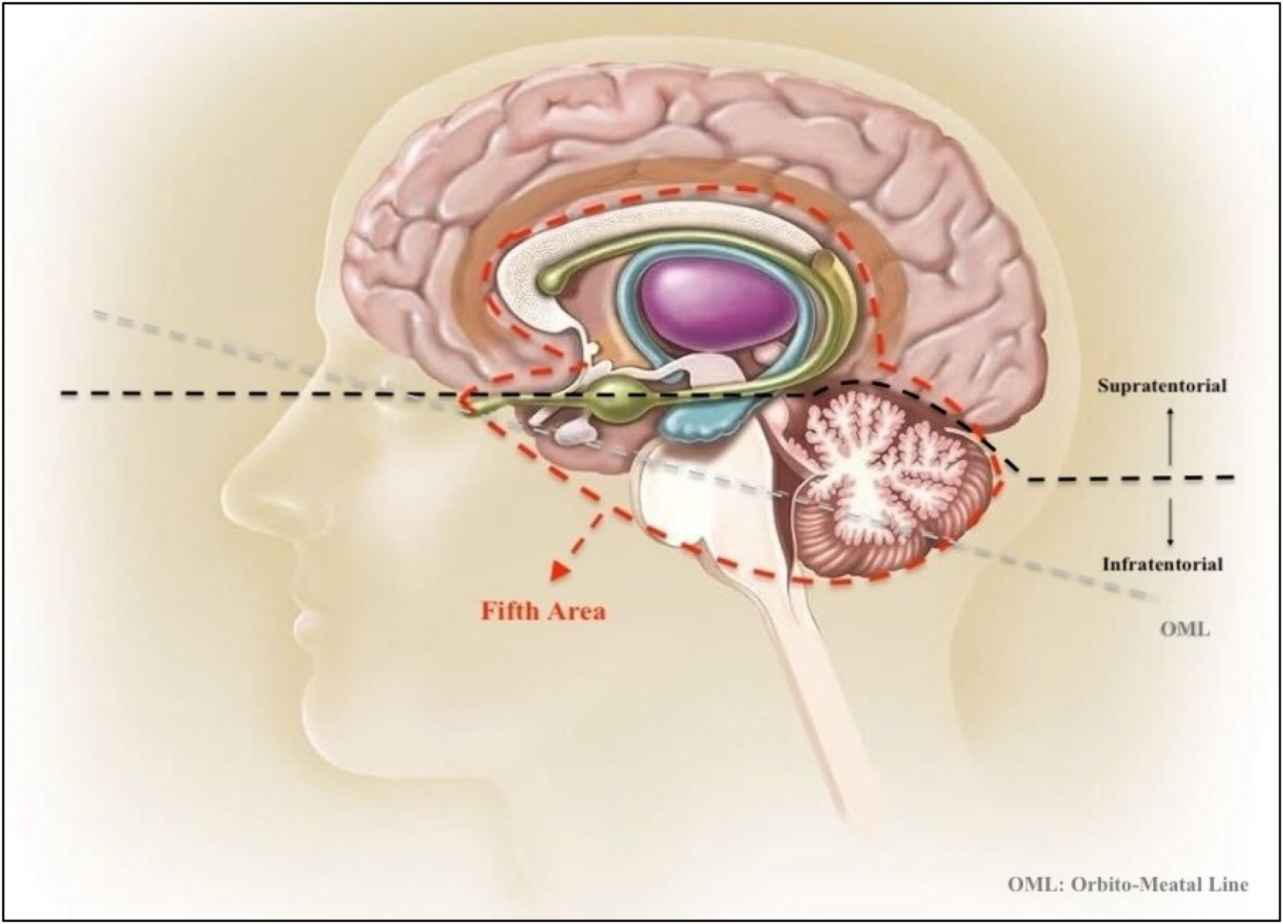
Illustration of the fifth area from a sagittal view of the brain. This illustration includes Cerebellum, Brainstem, Thalamus, Basal Ganglia, Corpus Callosum, Pons, Mesencephalon and Medulla Oblongata. The orbitomeatal line (OML) is a positioning line used in radiography of the skull, helping to orient the brain structures within the standard anatomical context. This figure provides a detailed view of the deep central regions of the brain, often involved in more complex neurological processes and pathologies.

Among the 36 patients with intracranial complications (ICC), subdural empyema (SDE) was detected in 25. Of these 25 patients, 16 did not undergo surgery, and 9 underwent surgical procedures. Patients with SDE were divided into two groups based on the presence of surgery and evaluated based on the anatomical location of the empyema, which was divided into 5 quadrants. It was found that the involvement of empyema in a single quadrant showed a significant difference between those who underwent surgery and those who did not (p: 0.012). Details are shown in the Table 4; Figures 4,5.

**Table 4:** Surgical interventions based on quadrant involvement.

Quadrant Involvement	Operated patients (*n* = 9)	Non-operated patients (*n* = 16)	*P*-value
Single quadrant	7/9 (78%)**	2/16 (13%)	**0.012** ^a^
Two quadrants	1/9 (11%)	6/16 (38%)**	
Four quadrants	1/9 (11%)	3/16 (19%)	
Five quadrants	0/9 (0%)	3/16 (19%)	
Isolated fifth area	0/9 (0%)	2/16 (13%)	

Bold values and ** indicate parameters with statistically significant differences between operated and non-operated patients (*p* < 0.05).

^a^ Pearson chi-square test was used, and significant difference was found between groups.

Upon reviewing the 25 patients with SDE, 5 were infratentorial, and 20 were supratentorial. Of the 5 infratentorial cases, 2 were exclusively infratentorial, and 3 were both infra- and supratentorial. None of the 5 patients with infratentorial empyema underwent surgery.

This analysis provides insights into how the extent of anatomical involvement may influence the decision for surgical intervention in patients with intracranial complications (ICC). The significant difference observed in single quadrant involvement suggests a higher tendency for surgery when SDE is localized, as indicated by the marked difference in surgery rates between the groups (78% vs. 13%).

## 4 Discussion

Prompt diagnosis and immediate treatment of acute bacterial meningitis (ABM) are critical for patient survival. In particular, early detection and surgical management of subdural empyema (SDE)—a neurosurgical emergency that can arise secondary to bacterial meningitis—are essential to reduce morbidity and mortality (7, 12–15). Delays in surgical intervention are associated with mortality rates exceeding 90% (16), whereas intervention within 72 h of symptom onset lowers the risk of disability to 10%, compared to 70% when delayed beyond 72 h (17).

Yılmaz et al. reported that all 28 children with SDE secondary to meningitis underwent decompressive craniotomy or burr hole procedures, while Kanu et al. performed surgery on 17 infants with complicated meningitis (18, 19). The average time to surgery was one to two months in these studies, with late presentations leading to 100% surgical intervention and severe neurological sequelae. These findings highlight the devastating impact of delayed recognition and treatment, particularly in resource-limited settings (20).

To improve early identification, several studies have proposed scoring systems to predict poor outcomes and identify patients at high risk for intracranial complications (ICCs), including SDE (10, 13). These efforts support closer monitoring in neurocritical care units for selected pediatric patients (10, 21).

Young age, particularly under one year, is a well-established risk factor for ICCs such as hydrocephalus, subdural effusion, SDE, seizures, and hearing loss (22). Subdural effusion occurs in approximately 40%–60% of infants with bacterial meningitis and may progress to SDE through secondary infection (23, 24). Immature immune responses and the vulnerability of the developing brain may contribute to the severity of outcomes (9). Additionally, male sex has been associated with an increased risk of SDE (18).

In our study, consistent with previous findings, ICCs were significantly more frequent in children under two years of age (*p* = 0.041). Notably, 5 of the 9 operated patients (55.5%) were younger than six months, emphasizing the need for heightened clinical vigilance and early intervention in this age group.

Previous studies have shown that high cerebrospinal fluid (CSF) protein and low glucose levels are associated with a greater risk of neurological sequelae, including hearing loss, epilepsy, and death (11, 13, 25). For example, CSF glucose below 1.5 mmol/L (26) or 40 mg/dl (25) has been linked to a 41.7% risk of sequelae. While these parameters have been used to predict complications, they do not differentiate which patients with ICCs will ultimately require surgery (21). Our study aims to fill this gap by evaluating CSF and serum markers alongside imaging findings to better predict the need for surgical intervention.

Elevated CSF protein levels have been associated with an intensified inflammatory response and are considered a risk factor for neurological sequelae (13, 27). Similarly, decreased CSF glucose reflects both the virulence of pathogens and the extent of brain damage, correlating with poorer prognosis (28). Several authors suggest that abnormalities in CSF protein and glucose not only indicate risk for neurological complications but may also signal the development of intracranial complications (e.g., SDE) in bacterial meningitis (7, 21, 29).

Previous studies have reported significantly abnormal CSF protein and glucose levels in patients who required surgical treatment for SDE (1, 30). Notably, Yalçınkaya et al. (21) proposed the CSF protein/glucose ratio as a screening tool, identifying a cut-off of 4.65 with 100% sensitivity—below which no patients developed SDE. In our study, a much higher ratio of 18.9—approximately four to five times that threshold—was strongly associated with surgical intervention, warranting serious clinical attention.

We found a significant difference in the CSF protein/glucose ratio between the non-operated (Group 1A; *n* = 27, mean 28 ± 62) and operated (Group 1B; *n* = 9, mean 189 ± 227) groups (*p* = 0.011). CSF glucose levels were also significantly lower in Group 1B (*p* = 0.049). While CSF protein levels did not reach statistical significance (*p* = 0.11), the near threefold increase observed in operated patients is notable and may be attributed to the limited sample size.

To further explore predictive performance, we conducted ROC analysis. A protein/glucose ratio cut-off of 18.9 yielded 77.8% specificity for identifying patients needing surgical intervention. A CSF glucose cut-off of 6.75 mg/dl provided 94.4% specificity, and a CSF protein cut-off of 304.5 mg/dl was associated with ICC development in 72.2% of patients.

Serum inflammatory markers such as CRP and WBC were also examined, as previous studies have linked them to the development of SDE (31, 32). While a WBC count >12,000 was significantly associated with ICC development (*p* = 0.010), it did not distinguish between operated and non-operated groups. Sequential measurements (day 3 and 4 CRP) can predict significant neurological sequelae, highlighting its importance in prognosis and clinical outcomes (33, 34). In contrast to WBC, CRP levels were significantly higher in Group 1B (226 ± 94) compared to Group 1A (63 ± 105) (*p* < 0.001). A CRP cut-off of 150.3 mg/dl was predictive of surgical need, with a specificity of 88.9%.

Our findings suggest that while WBC count >12,000 may indicate risk for ICCs, CRP levels are more reliable in predicting surgical necessity. These results underscore the importance of early LP and communication with families, particularly when lumbar puncture is delayed or refused. In such cases, CRP levels >150.3 mg/dl should prompt urgent neuroimaging, ideally with gadolinium-enhanced MRI, and immediate evaluation of CSF parameters to guide early intervention.

A CRP level above 150.3 mg/dl, a CSF protein/glucose ratio of 18.9, and a CSF glucose level of 6.75 mg/dl collectively indicate a high bacterial load and extensive infection—strong predictors of the need for surgical intervention. When all three parameters are present, they should be evaluated jointly as critical red flags in the management of complicated meningitis. Such cases warrant urgent surgical assessment.

In subdural empyema (SDE), particularly among neonates and young infants, clinical signs are often vague and nonspecific (35), complicating early diagnosis. Imaging thus becomes essential (6, 7, 14, 18). However, conventional CT scans may fail to detect relevant abnormalities. Studies report that up to 63% of CT scans in bacterial meningitis patients can appear normal, and CT is often inadequate in identifying cerebral herniation or localized fluid collections (36). In suspected cases of SDE, gadolinium-enhanced MRI remains the gold standard, as it is more sensitive in detecting small or localized collections (5, 36, 37). When venous sinus thrombosis is also suspected, MR venography may be required for comprehensive assessment (38).

In our cohort with ICC (Group 1, *n* = 36), 44% (16/36) had normal CT scans within the first 24 h. These unremarkable imaging results led to delays in obtaining gadolinium-enhanced MRI and initiating timely intervention. Among these 16 patients, 14 (87%) developed neurological sequelae, and 5 eventually required surgery. It appears that normal initial CT findings, coupled with high initial GCS scores (e.g., GCS 15) and subtle clinical presentations, may have contributed to underestimation of disease severity. Therefore, we emphasize that physicians should remain highly vigilant and pursue early MRI in the presence of abnormal CRP and CSF markers—especially when the “3 + parameters” are observed—even if initial imaging or clinical findings appear benign.

In resource-limited settings, late hospital admissions and normal or delayed CT reports often result in underestimating the severity of bacterial meningitis. Additionally, refusal of lumbar puncture by families leads to both misdiagnosis and inadequate treatment. These patients often experience prolonged hospital stays and eventually require surgical intervention due to worsening neurological complications. Our findings highlight that delayed recognition and intensive care interventions—rather than late presentation alone—are critical contributors to adverse outcomes. As previously reported, outcomes following delayed surgical treatment are typically poor (18, 19). Thus, when all three parameters (CRP >150 mg/dl, CSF glucose <6.75 mg/dl, and protein/glucose ratio >18.9) are present, urgent surgical evaluation, early neurosurgical consultation, and timely admission to pediatric intensive care are essential.

Elevated intracranial pressure (ICP) is most pronounced during the first 72 h of meningitis. If not controlled through aggressive medical treatment by days 3–5, it may lead to irreversible neurological damage or death (39, 40). Therefore, many experts recommend admitting high-risk patients to neurocritical care units during this early window for intensive monitoring (8, 13, 41), repeat imaging, and multidisciplinary decision-making to prevent poor outcomes (8, 10, 29, 30). Such patients should be managed by a coordinated team including pediatric intensivists, neurosurgeons, neurologists, infectious disease specialists, and rehabilitation experts (8).

In our study, we aimed to identify patients at surgical risk regardless of their clinical condition—including GCS scores, sepsis, shock, respiratory or cardiac arrest, intubation, or seizures. The presence of three or more positive parameters (3+) was found to significantly predict the need for surgical intervention. Based on this, we propose that all patients meeting this threshold should be admitted to the Pediatric Intensive Care Unit (PICU) for the first five days, even if they appear clinically stable. Only in this setting can aggressive treatment, close neuroimaging surveillance, and continuous monitoring be ensured to reduce ICP, surgical risk, and neurological sequelae.

In our cohort, nine patients required surgery between days 6 and 19 following diagnosis. This delay was primarily due to reassuring GCS scores, subtle clinical signs in infants, normal CT findings, and earlier misdiagnoses, which led to suboptimal antibiotic use. These factors likely masked the true severity of disease and prevented timely intervention. In patients meeting the 3+ criteria, regular and early imaging is vital—particularly as SDE-related complications such as sinus venous thrombosis, infarction, hydrocephalus, and ventriculitis can further elevate ICP (15, 32, 42). Notably, all nine patients who underwent surgery also had one or more of these complications.

To enhance early recognition of high-risk cases, we analyzed the anatomic distribution of empyema in patients undergoing initial MRI. Brain regions were divided into five quadrants, and in the surgical group (Group 1B), empyema were localized to a single quadrant in 7 of 9 cases (77.8%). In contrast, among the non-operated group (*n* = 16), only 2 cases (12.5%) had single-quadrant involvement—a statistically significant difference (*p* = 0.012). Although the literature associates intracranial mass lesions such as tumors and abscesses with elevated ICP, the effect of empyema size and distribution on ICP is less clearly defined. Our findings suggest that even small-volume empyema may exert considerable pressure when confined to a single anatomical region. This may be explained by the physical principle that pressure (P) equals force (F) divided by area (A). Given the same mass (force), a smaller area leads to higher pressure:

$$p=\frac{\;f}{a}$$

[Pressure is defined as force per unit area. It is expressed as \(P\) for pressure in pascals, \(F\) as the force in newtons, and \(A\) as the area in square meters.]

Thus, an empyema localized to a smaller surface may exert greater mechanical pressure on adjacent brain tissue than a more diffusely distributed one. While this remains hypothetical, our results support the notion that both the **biochemical severity (3+ parameters)** and **anatomic concentration** of the empyema contribute to surgical risk and worse neurological outcomes. Further studies are warranted to validate this model and improve surgical risk stratification.

### 4.1 Limitations

This study has several limitations. First, its retrospective design restricts the ability to control for confounding variables and may introduce information bias. Second, as it was conducted at a single tertiary care center, the findings may not be generalizable to other institutions or populations. Third, despite covering a 10-year period, the overall sample size—particularly within the surgical subgroup (*n* = 9)—was relatively small, limiting statistical power and external validity. Additionally, variability in treatment initiation timing, antibiotic regimens, and imaging practices may have influenced clinical management and outcomes. Furthermore, although surgical decisions were made based on consistent clinical and radiological criteria, such as the presence of empyema, abscess, midline shift, or signs of elevated intracranial pressure, the absence of a universally standardized surgical risk assessment tool introduces a degree of subjectivity that is inherent to retrospective neurosurgical evaluations. Nonetheless, the primary aim of our study was to identify early laboratory parameters—based on initial blood and CSF findings—that could assist clinicians in recognizing pediatric patients at risk for severe intracranial complications. We believe our findings offer valuable insights, but future prospective, multicenter studies using standardized clinical protocols and objective surgical criteria are needed to validate and expand upon these results.

## 5 Conclusion

Our findings suggest that the presence of three specific parameters—CRP >150 mg/dl, CSF glucose <6.75 mg/dl, and a CSF protein/glucose ratio >18.9—strongly indicates the need for surgical intervention in complicated bacterial meningitis. In such cases, immediate MRI is recommended to evaluate for intracranial complications, particularly subdural empyema (SDE). If the empyema is localized to a single quadrant, early neurosurgical consultation is essential. These high-risk patients should be managed in a pediatric intensive care unit (PICU) under multidisciplinary supervision to ensure timely diagnosis, appropriate intervention, and improved outcomes.

## Data Availability

The raw data supporting the conclusions of this article will be made available by the authors, without undue reservation.
